# High Q-contrast terahertz quantum cascade laser via bandgap-confined bound state in the continuum

**DOI:** 10.1515/nanoph-2024-0757

**Published:** 2025-05-01

**Authors:** Hanyu Liu, Jieyuan Cui, Qian Wang, Lianhe Li, Alexander Giles Davies, Edmund Harold Linfield, Qi Jie Wang

**Affiliations:** Centre for OptoElectronics and Biophotonics, 54761 School of Electrical and Electronic Engineering & The Photonics Institute, Nanyang Technological University, 639798, Singapore, Singapore; Institute of Materials Research and Engineering, Agency for Science, Technology and Research (A*STAR), 2 Fusionopolis Way, #08-03, Innovis, 138634, Singapore, Singapore; National Centre for Advanced Integrated Photonics (NCAIP), 50 Nanyang Avenue, S2-B3-01, 639798, Singapore, Singapore; School of Electronic and Electrical Engineering, University of Leeds, Leeds LS2 9JT, UK; School of Physical and Mathematical Sciences, 54761 Nanyang Technological University, 637371, Singapore, Singapore

**Keywords:** bound state in the continuum, photonic crystal, terahertz, Q-contrast

## Abstract

Photonic bound states in the continuum (BICs) are optical modes that remain highly localized despite co-existing with radiating waves in the continuum, attracting considerable attention for both fundamental studies and technological innovations. Conventional single-mode BIC lasers predominantly focus on maximizing the Q-factor of a specific mode, often overlooking the critical role of Q-contrast – the difference in Q-factors between the highest-Q BIC mode and competing modes – which is crucial for achieving stable single-mode lasing. In this study, we present a compact, high Q-contrast BIC laser, enabled by strategically optimizing the alignment of the TM_1_ band of the core domain with the shell domain to confine the high Q-factor mode within the core. Using a quantum cascade laser chip operating in the terahertz (THz) regime, this design achieves a Q-contrast ratio of approximately 2.3, resulting in stable single mode lasing across the dynamic region with a side-mode suppression ratio of ∼20 dB. These findings underscore the pivotal role of Q-contrast in photonic lasers, with promising implications for applications in THz lasers, sensors, harmonic signal generators and modulators.

## 1 Introduction

Bound states in the continuum (BIC) represent a fascinating class of phenomena wherein localized wavefunctions exist in an otherwise continuous spectrum without radiating energy [1], [2], [3]. Originally proposed as a mathematical concept by von Neumann and Wigner [4], BICs have been widely observed across various fields including electromagnetic waves, quantum mechanics, water waves and acoustic waves [3], [5], [6], [7], [8], [9], [10], [11], [12], [13], [14], [15], [16], [17], [18], [19], [20], [21], [22], [23], [24], [25], [26], [27]. In particular, BICs in photonic crystals (PhCs) have garnered significant attention due to their extreme high-Q resonances, design versatility and spectral scalability through cavity size adjustments [8], [26], [28], [29], [30], [31], [32], [33], [34], [35], [36]. Theoretically, symmetry-protected BIC modes located at the Γ point and have theoretical infinite high Q-factor due to symmetry mismatch with free space, making them ideal for active laser devices that require single-mode operation, low thresholds and high directionality [3], [5].

Nevertheless, most BIC lasers reported to date necessitate relatively large cavities in comparison to their lasing wavelength to achieve good mode confinement [37], [38]. Smaller PhC cavities are typically prone to significant transverse leakage, resulting in low Q-factor and higher threshold which might even prevent stimulated emission or stable single mode lasing, especially in the terahertz (THz) regime [34], [39], [40]. Various attempts have been made to address these challenges. In 2022, Chen et al. developed a mini-BIC by incorporating a photonic bandgap mirror around the mini-BIC to suppress transverse leakage, achieving a high Q-factor within a compact modal volume [41]. Shao et al. and Han et al. reported topological bulk lasers by confining a bulk state of a topologically photonic crystal within a trivial photonic crystal. This band-inversion-induced confinement introduces a novel lasing mode selection mechanism and controls emission directionality [42], [43]. Despite these advances, existing works primarily focus on maximizing the Q-factor, rather than the Q-contrast between the fundamental and higher-order modes. The Q-contrast plays a crucial role in ensuring a stable single mode laser, as once the pumping power reaches the threshold, higher-order modes also can gain sufficient amplification to lase, competing with the fundamental mode and potentially disrupting single-mode operation [44].

Here, we experimentally demonstrate an electrically pumped BIC laser with high Q-contrast through precise band engineering in a bandgap-confined hetero-photonic structure, implemented on a THz quantum cascade laser (QCL) platform. By positioning the TM_1_ mode of the core domain within the middle of the shell domain bandgap, only the symmetry protected BIC at the Γ point in core domain survives and shows extreme high Q-factor as well as a high Q-contrast of approximately 2.3. The side mode suppression ratio (SMSR) is ∼19 dB and the pumping area of the cavity is around 6*λ* ^2^. In contrast, the BIC modes without shell domain protection present low Q-factors and strong side mode emission.

## 2 Results

**Figure 1:**
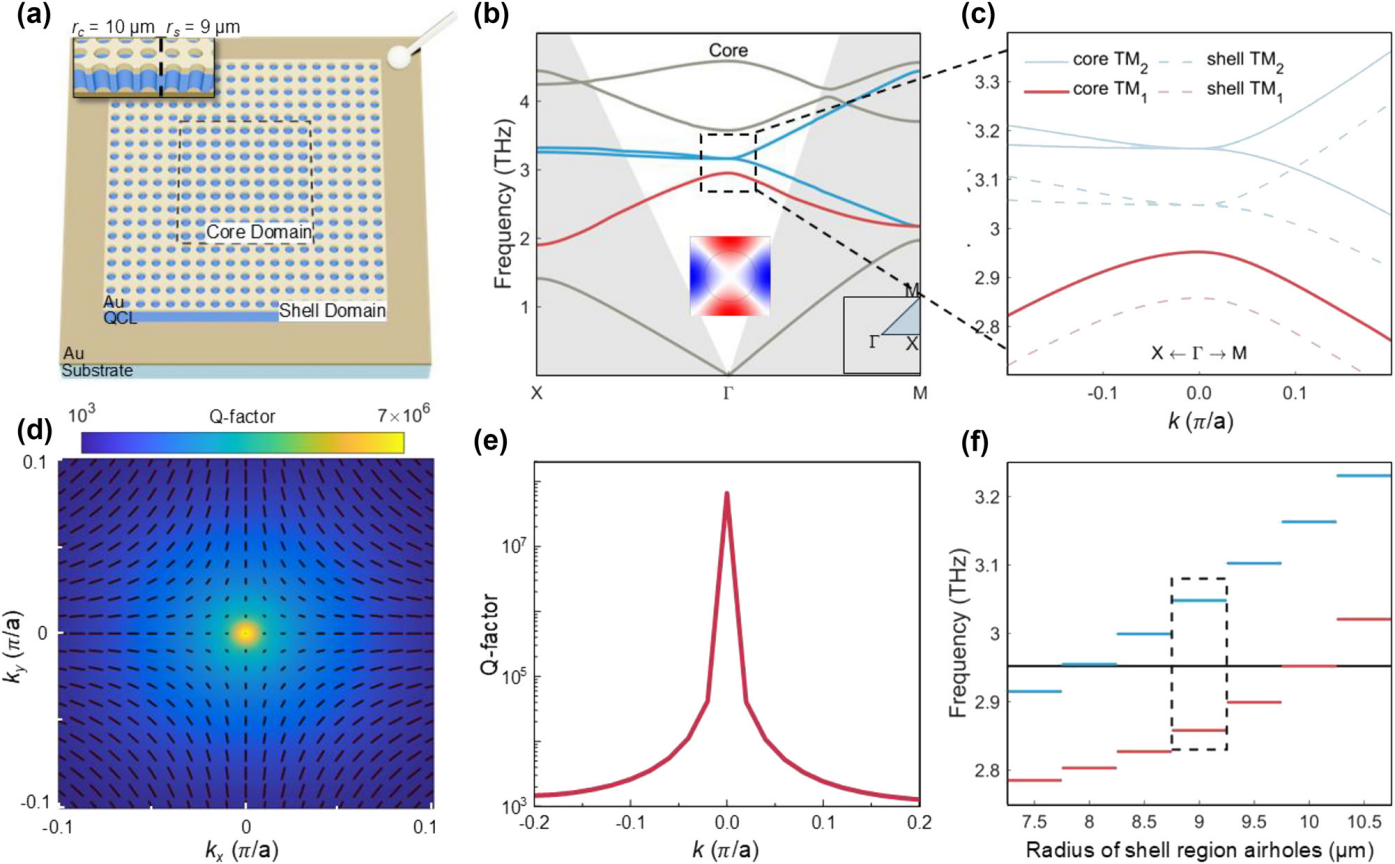
Design of the core–shell protected bound state in the continuum (BIC) photonic laser. (a) Schematic of the electrically pumped core–shell structure BIC photonic laser. The core domain with 9 by 9 periods is highlighted by the dash line and surrounded by shell domain. From top to bottom the layers are Au/QCL wafer/Au/n-type GaAs substrate. The top and bottom Au layers act as electrodes. Inset, cross-section of the BIC laser with Au/QCL layers are etched through. (b, c) Band structure of the modes in core domain calculated by 3D finite element simulation. In this work, focus is mainly put on the TM_1_ band highlighted by the red color. The grey shadow areas represent the region below the light line. The band structure of the modes in the shell domain (dotted lines) is also shown in Figure 1(c). The band structure of the shell domain is similar to that of the core domain (solid lines) but with frequency shifted. (d) 2D map of the Q-factor in momentum space around Γ point. Black dashed lines refer to polarization states. (e) Calculated Q-factors in momentum space with a divergent *Q* value at the *k* = 0 Γ point under infinite periodicity condition. (f) Illustration of the quasi-bandgaps of the shell domain with different airhole radius *r*_*s*_ and the TM_1_ mode belonging to the core domain. Black solid line refers to the eigenfrequency of the core domain at the Γ point, while the red and blue lines correspond to the shell bands. When *r*_*s*_ = 9 µm, the core band lies in the middle of the shell domain bandgap (highlighted by the black dashed circle). Other details on core–shell bandgap alignments are provided in Figure 3.

The proposed BIC laser cavity is coordinated in a two-dimensional (2D) square lattice with a lattice constant of *a* = 28 μm, thickness *d* = 18.3 μm, and refractive index *n* = 3.9. The photonic-crystal lattice is constructed by drilling cylindrical airholes through the QCL. The cavity comprises two domains, being the core domain with 9 by 9 periods and the surrounding shell domain, as illustrated in Figure 1(a). The only difference between the core and shell domain is the airhole radius, which is *r*_*c*_ = 10 µm for the core domain and *r*_*s*_ = 9 µm for the shell domain, respectively. The band structures for both domains share the same pattern, albeit with a frequency shift. We present the band structure for the core domain based on a unit cell with periodic boundary conditions. The TM_1_ mode highlighted in red color in the photonic band structure is selected to be the lasing mode (Figure 1(b)). In Figure 1(c), an enlarged image including the TM_1_ mode (shown as red lines) and two degenerated TM_2_ modes (shown as blue lines) is taken. The dashed lines refer to the band structure of the shell domain under the same 3D simulation. Figure 1(d) presents the simulated 2D Q-factor brightness map together with polarization distribution (black dashed lines) in momentum space. As illustrated, the polarization states exhibit a singularity at the center of the Brillouin zone, confirming it as a typical symmetry-protected BIC. The topological charge (*q*) was calculated to be *q* = +1, following the equation [6]:

$$q=\frac{1}{2\pi }\oint_{C}dk\cdot \nabla_{k}\phi \left(k\right),$$

where *C* is a counterclockwise closed loop surrounding the polarization field center and $\phi \left(k\right)=\arg\left[c_{x}\left(k\right)+ic_{y}\left(k\right)\right]$ represents the polarization angle at each wavevector ***k*** = (*k*_*x*_, *k*_*y*_). The symmetry-protected BIC mode occurs at the Γ point with theoretically infinite Q-factor, as shown in Figure 1(e). To optimize in-plane mode confinement of the symmetry-protected BIC mode in a miniaturized cavity, fine-tuning of the airhole radius in the shell domain are performed. Figure 1(f) presents the eigenfrequencies of bands at the Γ point for both the core and shell domain, with black line representing the TM_1_ mode of the core domain, and the red (blue) lines corresponding to the TM_1_ (TM_2_) modes of shell domain. Through careful design and manipulation, the relative position of the TM_1_ mode (core domain) within the bandgap of the shell domain can be well controlled, thereby confining the electric field to the center of the lattice. We observed optimal mode confinement and selectivity when the airhole radius of the shell domain was *r*_*s*_ = 9 µm, with the TM_1_ mode of core domain located at the center of the shell domain bandgap, as indicated by the black dashed square.

**Figure 2:**
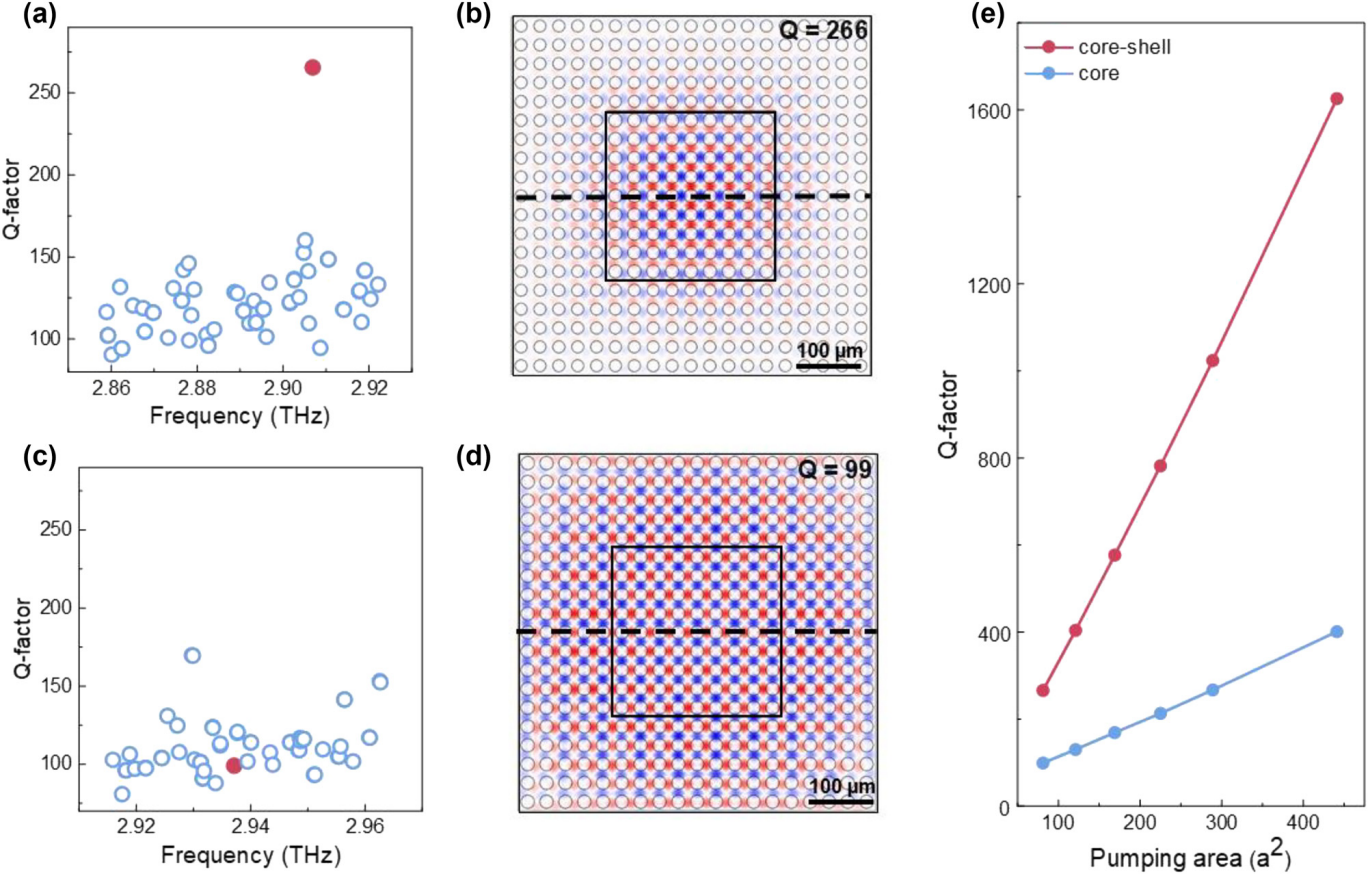
Comparison of cavity performance with and without shell domain modulation. (a, c) Calculated Q-factors of the eigenmodes for structure with shell (a) and without shell (d). (b, d) The electric fields (*E*_*z*_) of the lasing mode shown as the red dots in (a) and (d) correspondingly. In core–shell structure, only one single lasing mode with relatively high Q-factor exist, while in cavity without shell modulation, the Q-factor is lower and submerged in other bulk modes. (e) Q-factor as a function of pumping area for both core–shell structure and core domain. Compared to Figure 1(e), the Q factors are rather low due to scattering boundary conditions and finite periodicity.

The above numerical results are all based on single unit cell simulation with periodic boundary conditions. We also performed the full wave simulation based on real laser device configuration with finite size to demonstrate the importance of the shell domain in our design. We compared the performance of cavities with and without the shell modulation. As shown in Figure 2(a), the TM_1_ lasing mode marked as red solid dot in the core–shell cavity is significantly higher (exceeding 250) than other side modes. As a comparison, the Q-factor of the core-only structure is only ∼100 and submerged in other bulk modes (Figure 2(c)). Figure 2(b) and (d) illustrated the electric field (*E*_Z_) distribution in these two cavities. In the core–shell structure, the BIC mode is effectively confined within the pumped region, with the electric field intensity (extracted along the black dashed cutline in Figure 2(b)) showing a rough Lorentzian shape (Figure S2(a) in Supplementary Information). The sharp intensity drop at the domain boundary in the cavity demonstrates effective mode confinement. In contrast, for the cavity without shell modulation, the mode extends substantially out of the central region, exhibiting a typical Gaussian-shaped electric field distribution (Figure S2(b)). The huge side leakage leads to weaker mode confinement and a significantly lower Q-factor [37], [38]. This noteworthy difference arises from the mode mismatch between the M_1_ mode of the core domain and bandgap of the shell domain in the core–shell design, which effectively traps the lasing mode at the center of the cavity. As the pumping area increases, the Q-factor of the core–shell cavities shows a more pronounced enhancement that core-only structures, as demonstrated in Figures 2(e) and S3. It should be noted that the Q-contrast remains constant as the core domain enlarges, indicating no evident universal relationship between Q-contrast and core domain size, as illustrated in Figure S4.

**Figure 3:**
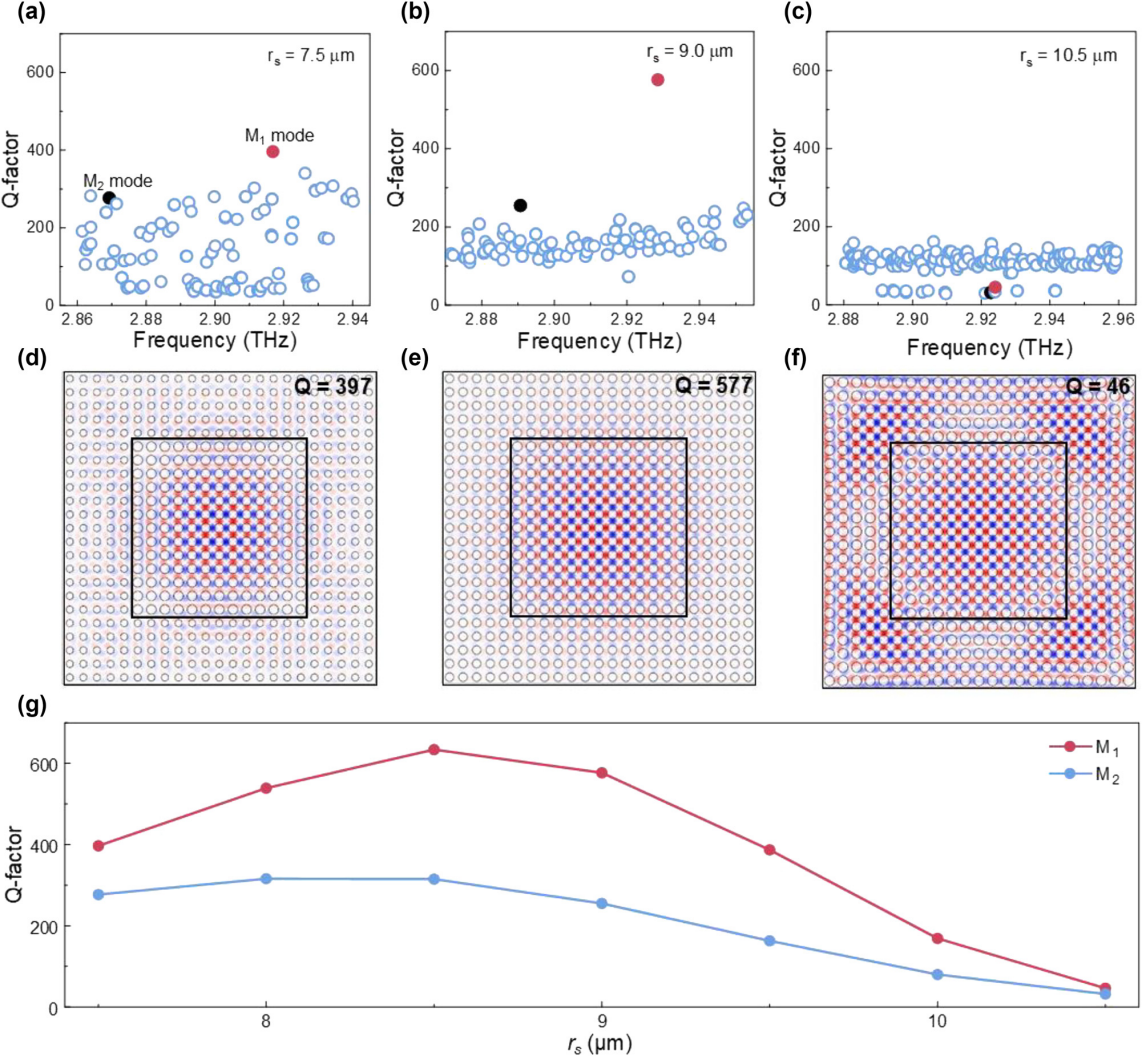
Selection of shell domain airhole radius. (a–c) Calculated Q-factors of the eigenmodes for core–shell structures with different shell airhole radius *r*_s_ of 7.5 µm, 9 µm and 10.5 µm. Red filled dots refer to the M_1_ modes and blue filled dots refer to one of the degenerated M_2_ modes. (d–f) The electric fields of M_1_ modes shown in (a), (b) and (c), respectively. (g) Q-factor as a function of shell airhole radius for M_1_ mode and one of the degenerated M_2_ mode.

Actually, to achieve stable single-mode laser operation, the Q-contrast between the target mode and other high-order modes is more critical than the absolute value of the Q-factor. We found that the choice of airhole radius in the shell domain can significantly impact both the Q-factor and side mode suppression ration (SMSR). Various airhole radii in the shell domain were compared, as shows in Figure 3(a)–(c), with values ranging from *r*_*s*_ = 7.5 µm to *r*_*s*_ = 10.5 µm accordingly. The devices all have a core domain of 13 by 13 periods surrounded by a shell domain of 5 periods. Specifically, we highlight the fundamental mode (M_1_) of the TM_1_ band (red solid dot) and neighboring higher order modes (M_2_, blue solid dot, degenerated hence here only M_21_ is shown for simplification, more details are given in Supplementary Information). As the shell domain airhole radius, *r*_*s*_, increases from 7.5 µm to 10.5 µm, the Q-factors of both modes initially rise and then fall, with the M_1_ mode showing a greater variation trend compared to the M_2_ mode. Both modes reach their peak Q-factors at *r*_*s*_ = 8.5 µm. Q-contrast between the two modes also follows a similar pattern, with a maximum value of approximately 2.3 occurring at *r*_*s*_ = 9 µm, where the TM_1_ band of the core domain roughly lies in the middle of the shell domain bandgap. The electric field distributions for the M_1_ modes at different airhole radii are presented in Figure 3(d)–(f), with the best confinement observed around *r*_*s*_ = 9 µm. Other radius outside this region (*r*_*s*_ = 7.5 µm to *r*_*s*_ = 10.5 µm) refers to even worse cavity performances and were not included here. We also note that despite the Q-factor of the cavity with *r*_*s*_ = 8.5 µm is slightly higher than that of *r*_*s*_ = 9 µm, the Q-contrast is lower, as detailed in the Supplementary Materials. Thus, the shell domain radius with *r*_*s*_ = 9 µm is selected for the experiments described below.

**Figure 4:**
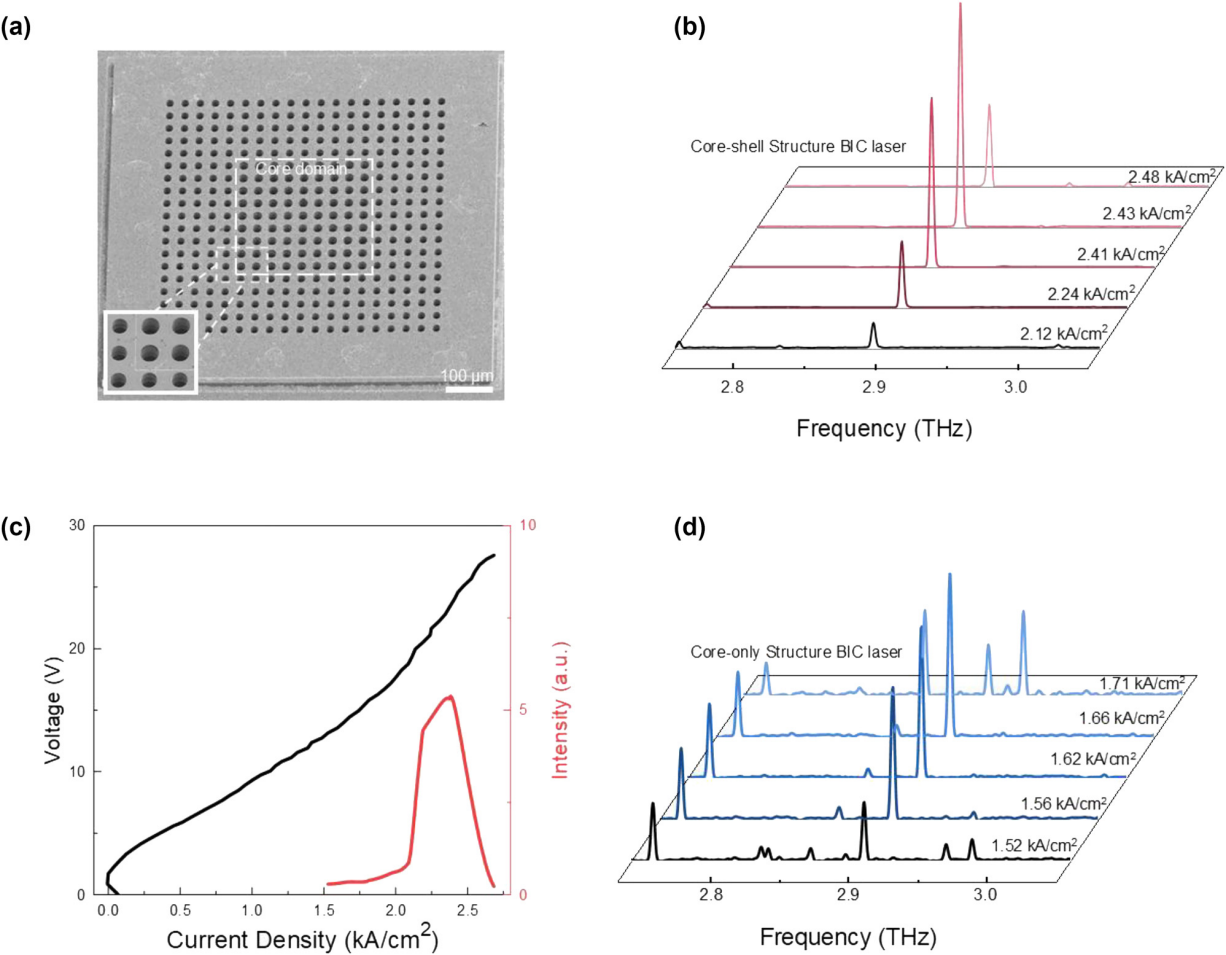
Characteristics of the core–shell structure BIC laser. (a) Scanning electron microscope (SEM) image of the fabricated core–shell structure BIC cavity. The periodicity of the core domain is 9 by 9 and is surrounded by the shell domain with 5 periods. Here *r*_*c*_ = 10 µm and *r*_*s*_ = 9 µm. Inset, a 2 by 2 periods of core domain (top right corner) surrounded by shell domain, where airholes in the core domain are slightly larger than those in the shell domain. (b) Emission spectra for BIC laser shown in (a) under different pumping current density. The highest output lasing intensity happens at 2.43 kA/cm2 and the highest Q-contrast happens at 2.41 kA/cm2. (c) Light-current-voltage curve of the core–shell structure BIC laser with 9 by 9 periods of core domain with *r*_*c*_ = 10 µm and *r*_*s*_ = 9 µm. The measurement was conducted at 9 K. (d) Emission spectra for BIC laser of 13 by 13 periods of core domain without shell protection for comparison with the proposed core–shell BIC laser. A typical multi-mode operation occurs as the current density increases, resulting in a low Q-contrast.

**Figure 5:**
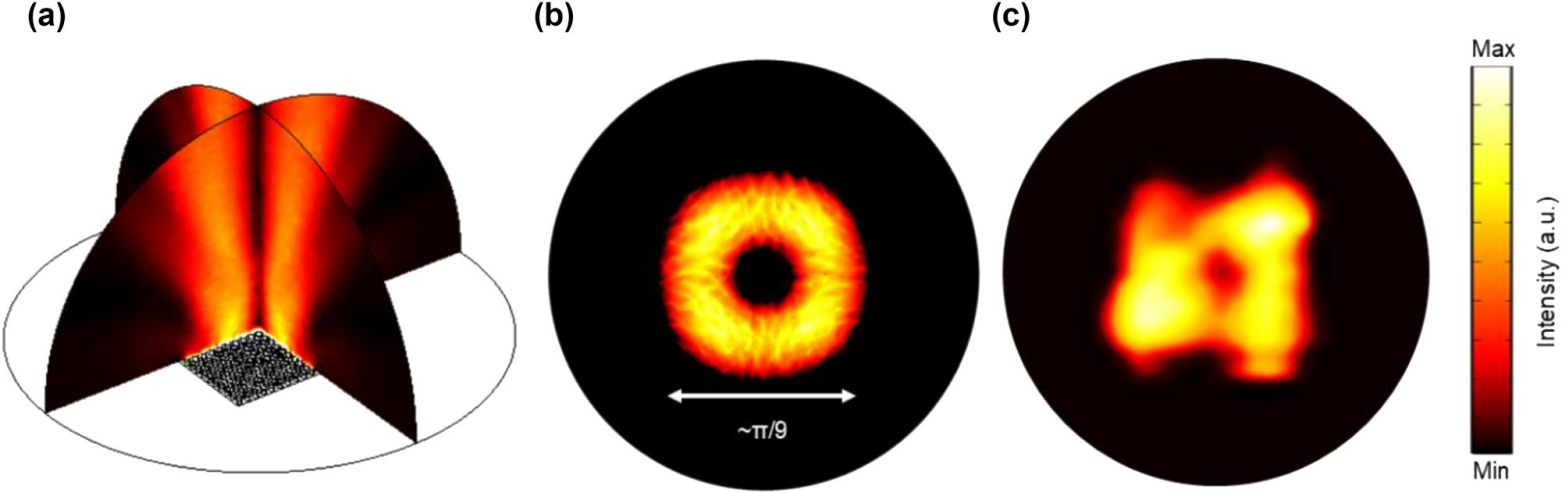
Calculated far-field image and measured far-field image of core–shell structure BIC laser. (a) Side view of calculated far-field image from *x*–*z* to *y*–*z* direction. A well-controlled divergence of the lasing beam ∼π/9 is observed. (b) Top view of the calculated far-field image, which exhibits the doughnut shape of a cylinder vector (CV) beam. (c) Measured far-field image of the core–shell structure BIC.

Figure 4(a) presents a scanning electron microscope (SEM) image showing the top view of the fabricated bandgap-confined BIC laser, consisting of 9 by 9 periods in the core domain and 5 periods in the shell domain. To achieve optimal lasing confinement, the air hole radius was set to be *r*_*c*_ = 10 µm for the core domain and *r*_*s*_ = 9 µm for the shell domain airholes. The lattice constant is *a* = 28 µm for the fabricated BIC laser. The emission spectra in Figure 4(b) indicate stable single-mode performance across the entire dynamic range. The lasing threshold is ∼2.1 kA/cm^2^, and the lasing output intensity increases as the pumping current rises. The output power reaches its maximum at 2.43 kA/cm^2^ and then drops down due to sub-band misalignment in QCLs. The characterized LIV plot is shown in Figure 4(c). For comparison, the emission spectra of another core-only device with 13 by 13 periods of core domain are shown in Figure 4(d), which exhibits a typical multi-mode operation with side modes increase severely. The highest SMSR estimated from the emission spectra for both devices are shown in Figure S8(a) and (b). For the core–shell device with 9 by 9 periods of core domain, highest SMSR of 19 dB occurs at current density of 2.41 kA/cm^2^. For the core-only device with 13 by 13 periods of core domain, highest SMSR of less than 4 dB occurs at current density of 1.62 kA/cm^2^. The highest SMSR estimated of a core-only device with 15 by 15 periods of the core domain has been added in Figure S8(c), which remains below 4 dB. The low SMSR observed in both core-only devices further confirm that without shell domain protection, multiple lasing modes occur and SMSR is noticeably lower than SMSR for BIC laser with shell domain protection, even with larger core domain. Figure S9(a) and (b) illustrates the measurement setup and the power output of a core–shell structure BIC device with 21 by 21 periods of core domain. To verify that the single-lasing mode originates from the theoretically predicted BIC mode by core–shell confinement, we compare the calculated far-field pattern with the experimentally measured far-field beam profile. Figure 5(a) demonstrates the side view of the calculated far-field pattern, indicating a small divergence angle of the lasing beam ∼π/9, with further explanation in Figure S10. Figure 5(c) presents the measured far-field emission from a top view, revealing a four-fold symmetric beam profile along with a central low-intensity doughnut shape region. This pattern closely matches the calculated far-field profiles from the top view shown in Figure 5(b), confirming the topological nature of the single-lasing mode. All the above results collectively demonstrate that our core–shell BIC mode laser is a promising candidate for achieving stable single mode THz lasing.

## 3 Discussion

In this work, we propose and experimentally demonstrate an electrically pumped THz laser utilizing a bandgap-confined symmetry-protected BIC design. By strategically constructing a shell domain surrounding the core domain, achieved through precise adjustments to the airhole radii in both domains, the fundamental BIC lasing mode in the core domain is effectively confined within the bandgap of the shell domain. Crucially, we enhance the Q-contrast between the fundamental lasing mode and other higher order modes by modulating the relative position of the target BIC mode within the bands of shell domain. This optimization enables stable single-mode operation, with an estimated SMSR of 19 dB, within a compact laser cavity. Our core–shell BIC laser exhibits promising potential for applications in molecular bio-sensing, wireless communication and various other endeavors within the THz frequency range.

## 4 Methods

### 4.1 Device fabrication

The BIC laser cavities were designed on a three well resonant-phonon GaAs/Al_0.15_Ga_0.85_As THz QCL wafer. To create an unpumped region, a SiO_2_ insulating layer was inserted between the top Au contact layer and the THz emission layer.

A 10 nm Ti layer followed by a 700 nm Au layer was sequentially deposited on the *n* ^+^ doped GaAs substrate and the THz QCL wafer using an electron-beam evaporator. The bottom Au contact layer is formed via Au–Au thermal-compression wafer bonding. To reduce the QCL buffer layer thickness to below 80 nm, a polishing instrument was utilized, and the residual GaAs substrate was removed through a wet etching process using citric acid/H_2_O_2_/H_2_O (92 g powder/30 ml/92 ml) and hydrofluoric acid (49 %) until the QCL active region was exposed.

To initiate the fabrication of BIC laser cavities, a 200 nm thick SiO_2_ layer was deposited to function as the insulating layer. Optical lithography was employed to define the pumping area. Reactive-ion etching (RIE) was then used to remove the SiO_2_ layer above the BIC laser core domain. Subsequently, the photonic crystal structure was defined through optical lithography on top of the QCL wafer. Contact layers (Ti/Au/Ti/SiO_2_, 20/350/20/710 nm) was then deposited and lifted-off atop the photonic crystal structure pattern. Inductively coupled plasma etching (ICP-RIE) was performed to dry-etch though the active region, while the remaining contact layer acting as a hard mask. A gas mixture of Cl_2_/BCl_3_/Ar = 4.5/4.5/4.5 standard cubic centimeters per minute was applied, which also consumed the top SiO_2_ layer. Following this process, the remaining Ti/Au layer would serve as the top contact layer for current injection. Bottom electrode was achieved by thinning the bottom host substrate to approximately 200 µm using a polishing instrument before depositing a Ti/Au layer (20/200 nm). The device was then packaged by cleaving the samples and indium-soldering onto a copper pedestal. For measurement, the device was wire-bonded and connected to a cold finger in a cryostat.

### 4.2 Characterization

The cryostat system containing the BIC THz lasers was maintained at a temperature of approximately 8.8 K. The lasers were pumped by an electrical pulse generator with 10 kHz repetition rate and a 500 ns pulse width for spectral measurements. Spectral resolution was set to 0.08 cm^−1^ and spectra were collected with a Bruker Vertex 80 Fourier-transform infrared (FTIR) spectrometer equipped with a room-temperature deuterated-triglycine sulfate (DTGS) detector.

### 4.3 Numerical calculation

All simulation results were generated using the commercial finite element method software COMSOL Multiphysics. The eigenfrequency solver was used to calculate band structures, eigenmodes and Q-factors at each *k*-point in the BIC photonic crystals through 3D simulations. The core and shell domain unit cells were represented by a cubic structure with a circular airhole of varying different radius drilled through the 18.3 µm-thick GaAs active region layer. Periodic boundary conditions were applied in the *x*–*y* plane. The refractive index of the active region layer was set to 3.9 and two perfect electric conductor layers were applied at the top and bottom of the active region layer to extract TM modes. In the full structure simulation, 9 by 9 periods of core domain were pumped and treated as lossless, while the surrounding shell domain was considered lossy, with an imaginary part of 0.016. To avoid back reflection of outgoing waves, scattering boundary conditions were implemented.

## Supplementary Material

Supplementary Material Details
