# Supplementary material for: High Q-contrast terahertz quantum cascade laser via bandgap-confined bound state in the continuum
Source: Nanophotonics. 2025 May 1;14(21):3433–41. doi: 10.1515/nanoph-2024-0757 (PMC12552860; doi:10.1515/nanoph-2024-0757)
Supplement: Supplementary file 1 — Supplementary Material Details [file j_nanoph-2024-0757_suppl_001.docx]

**Supplementary information**

**Hanyu Liu^1,2^, Jieyuan Cui^1^, Lianhe Li^3^, Alexander Giles Davies^3^,** **Edmund Harold Linfield^3^, Qian Wang^2^, Qi Jie Wang^1^***

^1^Centre for OptoElectronics and Biophotonics, School of Electrical and Electronic Engineering & The Photonics Institute, Nanyang Technological University, 639798, Singapore.

^2^Institute of Materials Research and Engineering, Agency for Science, Technology and Research (A*STAR), 2 Fusionopolis Way, #08-03, Innovis, 138634, Singapore.

^3^School of Electronic and Electrical Engineering, University of Leeds, Leeds LS2 9JT, UK.

**Email: *qjwang@ntu.edu.sg**

1. **Band structure of the utilized quantum cascade wafer**


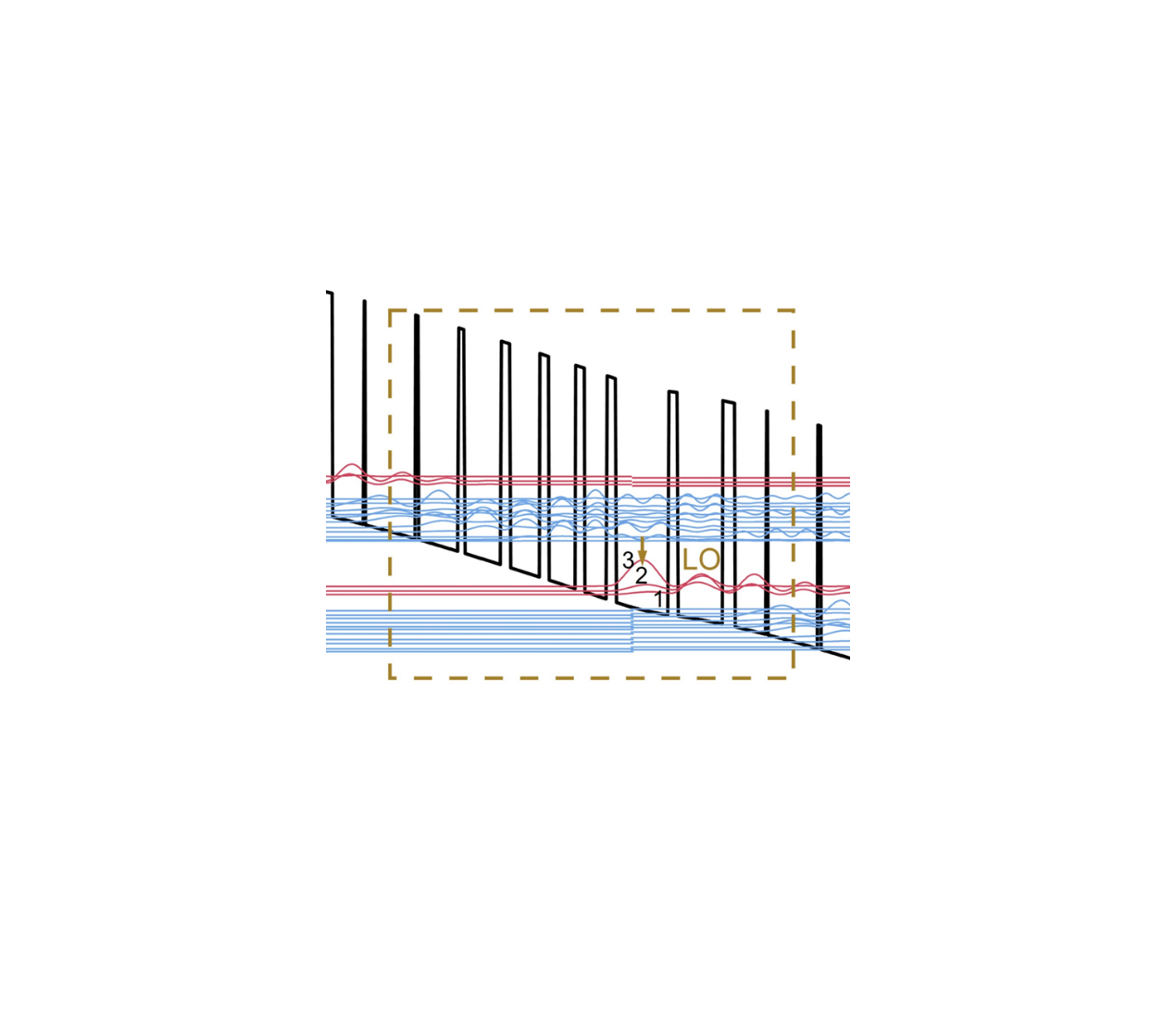


**Figure S1:** Conduction band profile and electron wavefunctions of the quantum cascade wafer we used in our BIC laser. The yellow dash box indicates a single period of the active region. The active region of the THz QCL consists of 95 repeating periods with alternating barriers (Al_0.15_Ga_0.85_As) and quantum wells (GaAs). A single period includes **1**/12.9/**2**/11.8/**3**/9.5/**3**/8.6/**3**/7.1/**3**/17/**3**/14.5/**4**/10.1/**0.5**/16.2, in which thickness is given in nanometers, each bold number refer to one Al_0.15_Ga_0.85_As barrier. The 17nm GaAs layer in the middle of one single period is n-doped with Si doing of 2.0ⅹ10^16^ cm^-3^. The electron wavefunctions along with the intersubband transition process is shown in Figure S1 with the use of tight-binding model.

1. **Mode Intensity Along Cutline for Core-shell and Core-only BIC Lasers**


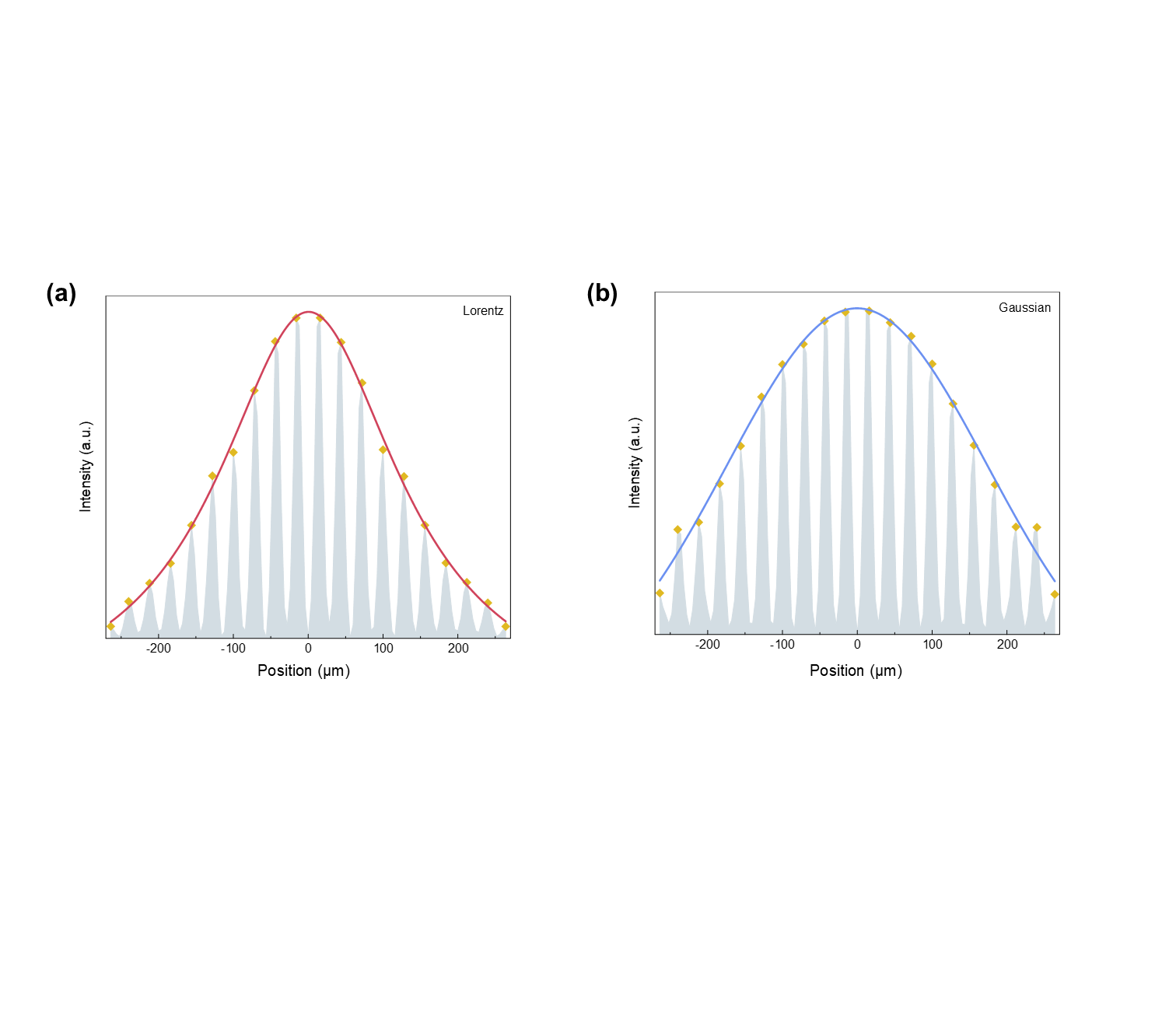


**Figure S2:** (a,b)Mode Intensity along the red and blue dash lines in Figure 2(b) and Figure 2(d) correspondingly. With the shell protection, the intensity along the dash line fits Lorentz curve; without shell protection, the intensity along the dash line fits Gaussian curve.

1. **Q-factor of core-shell and core-only BIC lasers with different core domain periodicities**

**
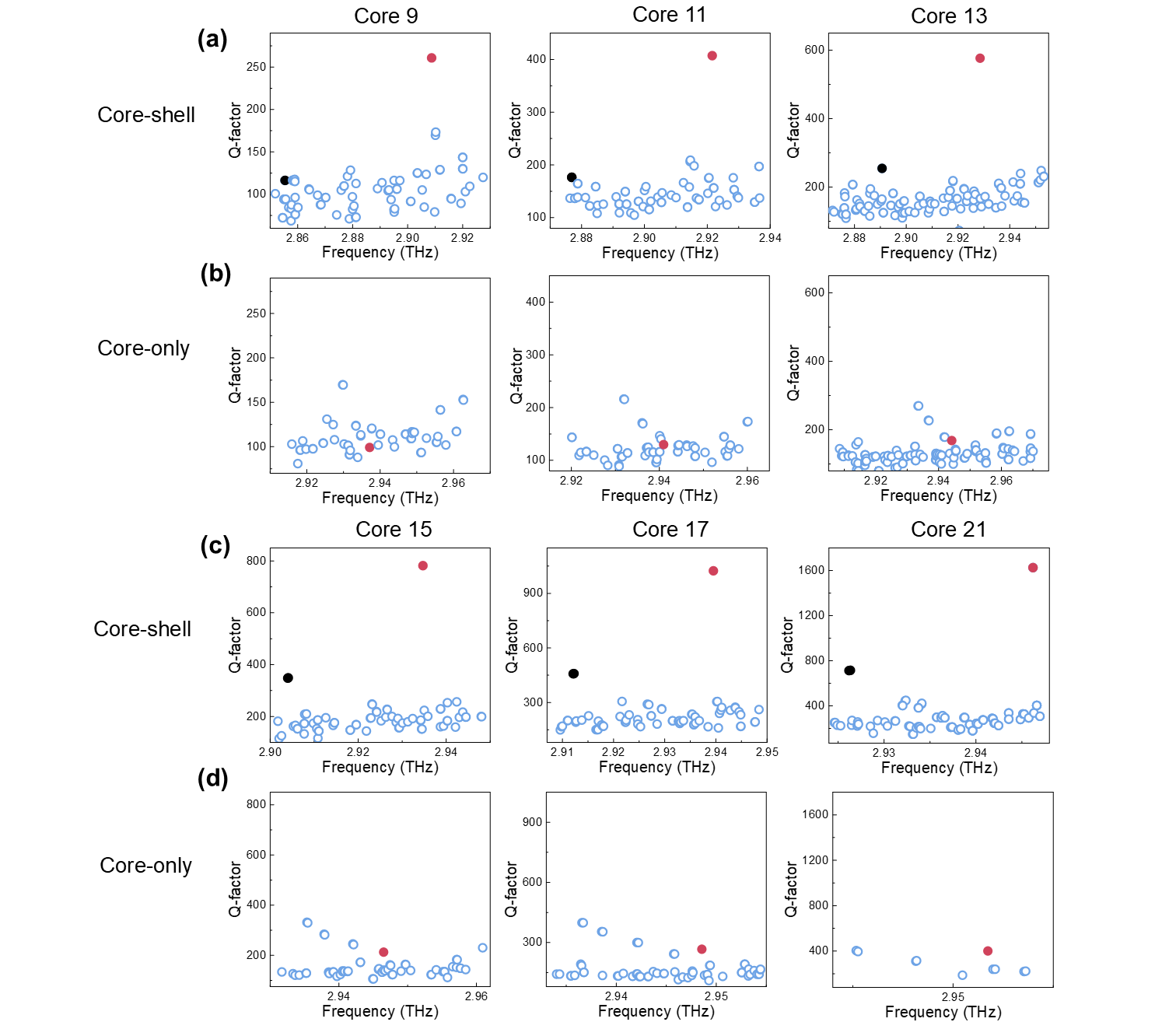
**

**Figure S3:** Calculated Q-factor for BIC lasers of different core periodicities with (without) shell domain. Core periodicity increases from 9 to 21, showing an increasing Q-contrast. Second-order lasing modes are marked as black solid dots.

**
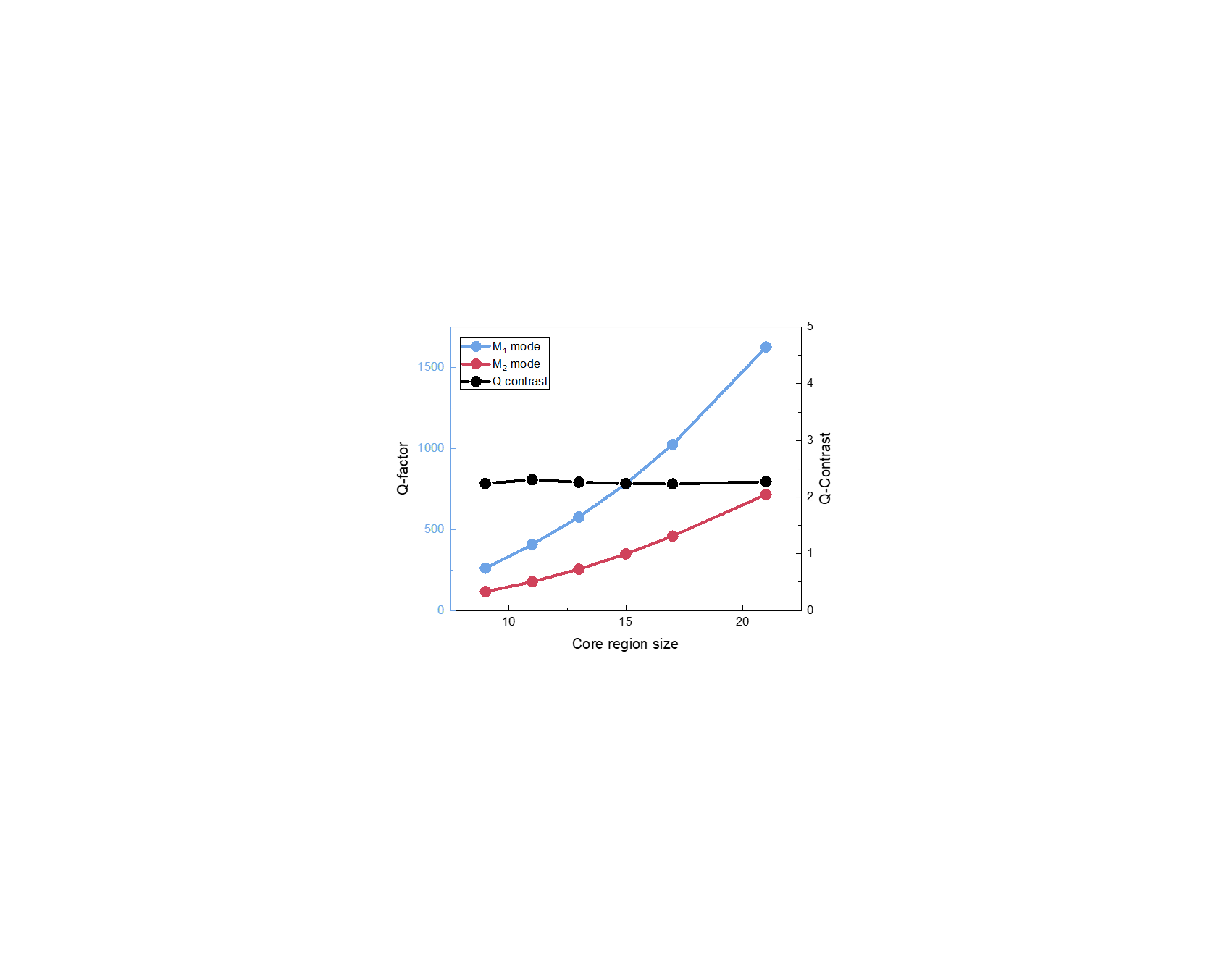
**

**Figure S4:** Calculated Q-contrast ratio for cavities with different core domain size.

1. **Side Mode Suppression Ratio (SMSR) for Lattice with Different Shell Domain Airhole Radius**


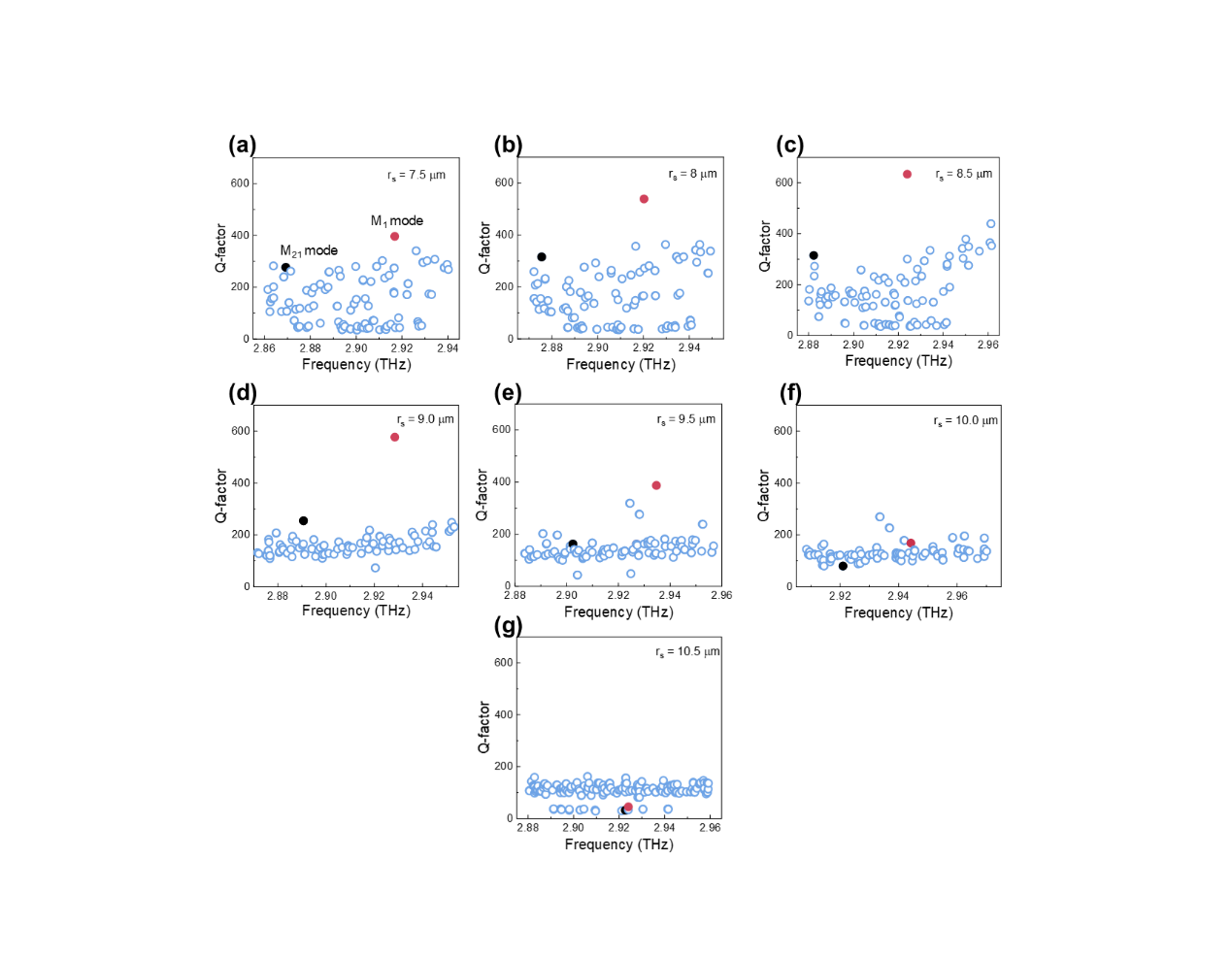


**Figure S5:** (a-g) Calculated Q-factors of the core-shell structure with different shell radius *r_s_* from 7.5 µm to 10.5 µm, with each sub-plot adding radius by 0.5 µm.


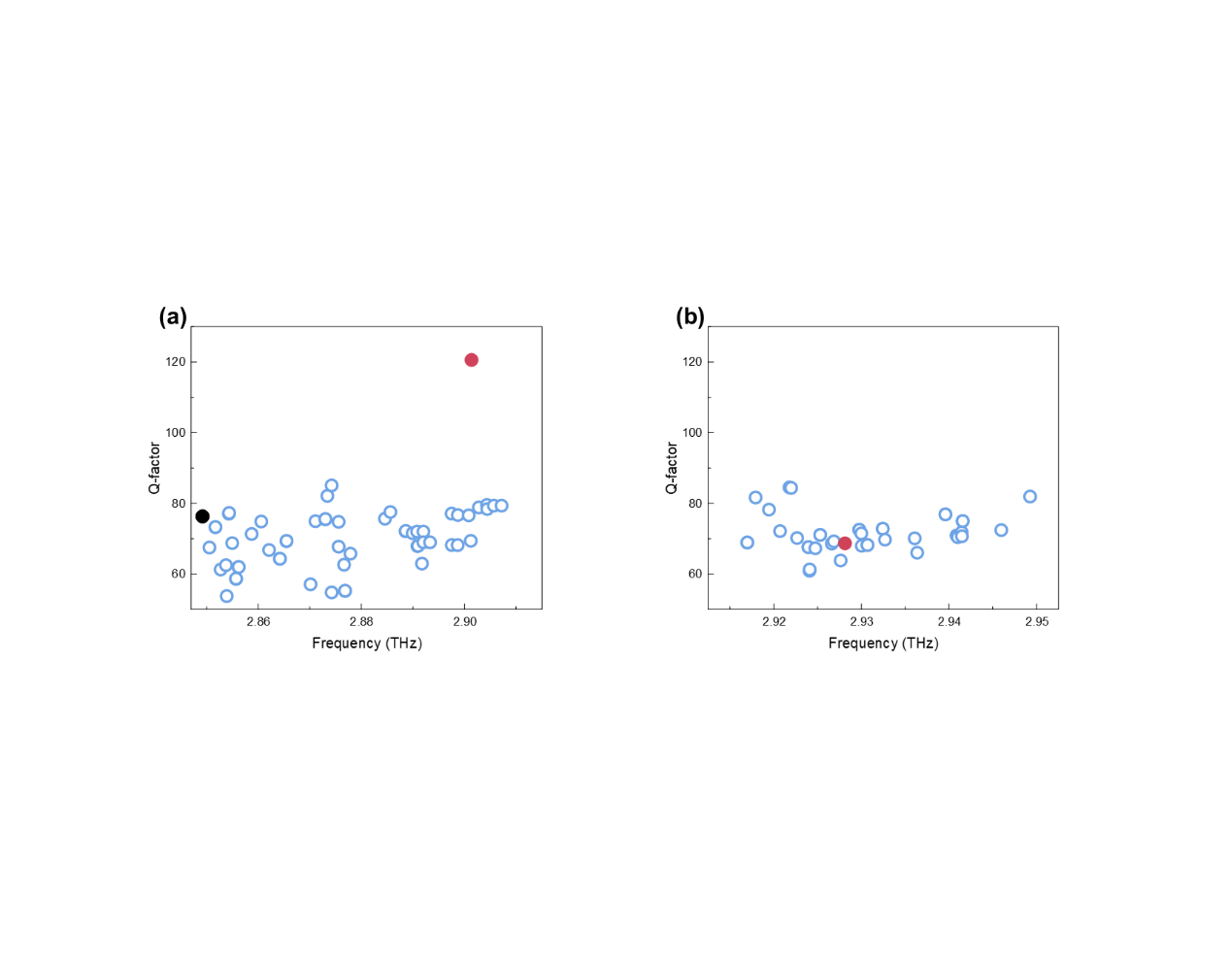


**Figure S6:** (a,b) Calculated Q-factors of the BIC laser of a 9 × 9 period core domain with (without) shell domain protection under Drude model conditions. For core-shell structure BIC laser, fundamental lasing mode (red solid dot) exhibits a Q-factor of ~121, while the second-order mode (black solid dot) has a Q-factor of ~76, yielding a Q-contrast of ~1.6. For core-only structure BIC laser, the Q-factor for the corresponding fundamental lasing mode is 68.


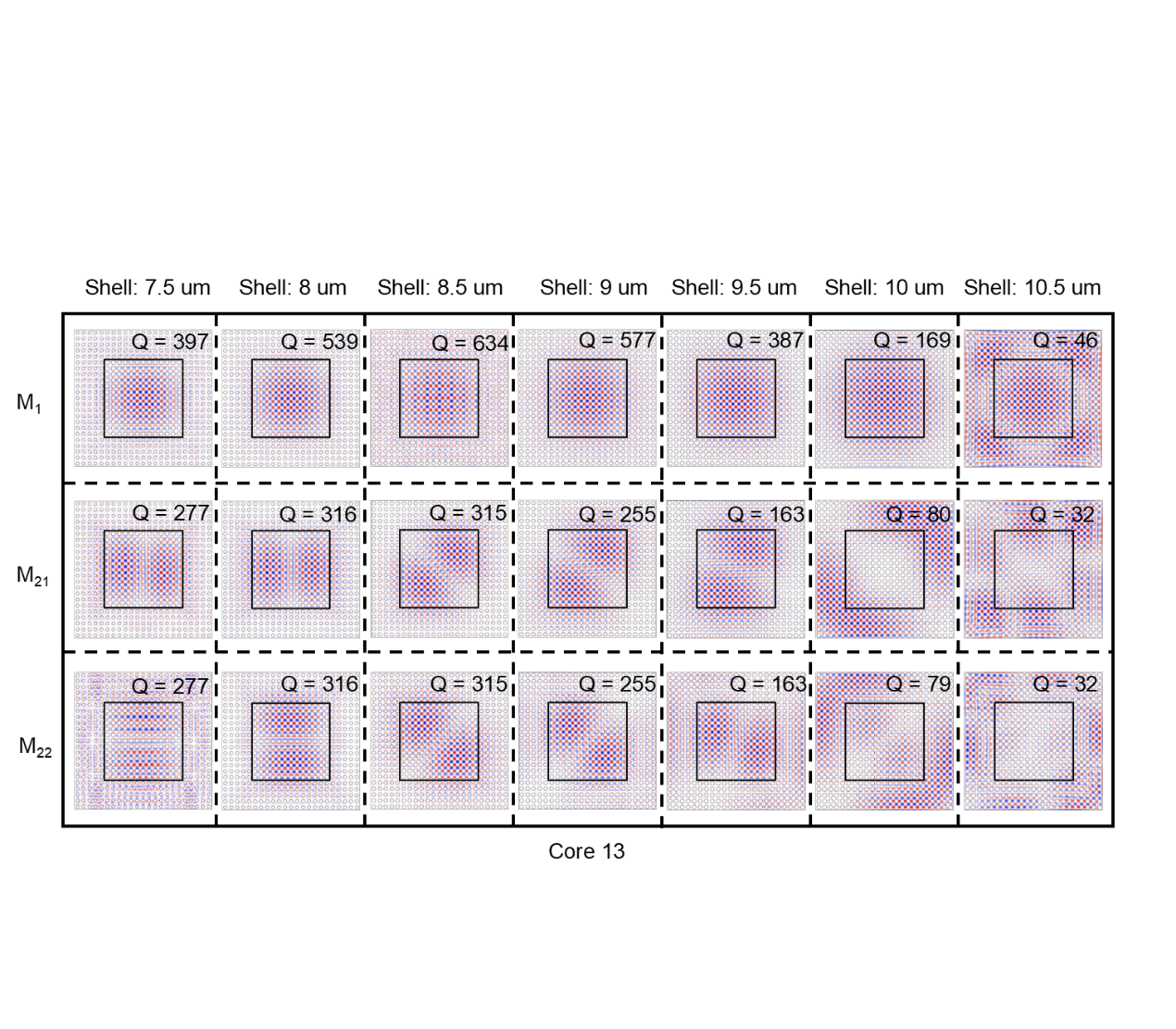


**Figure S7:** The electric fields of M_1_, M_21_ and M_22_ modes, as shown in Figure S3(a-g). The radius of shell domain airhole, *r_s_*, various from 7.5 µm to 10.5 µm to match the bandgap of the proposed core domain. Outside of this selected range, bandgap matching weakens significantly, leading to a substantial reduction in the Q-factor of the fundamental lasing mode compared to neighboring side modes. In Figure S5(a-g) red solid dots indicate M_1_ lasing mode, while blue solid dots represent one of the neighboring degenerated higher order M_2_ modes, specifically M_21_ mode. In Figure S7 we present the electric fields of M_1_, M_21_ and M_22_ mode along with their corresponding Q-factors. The electric field confinement of the M_1_ mode initially increases from *r_s_* = 7.5 µm, reaching its peak at *r_s_* = 9 µm, before decreasing at *r_s_* = 10.5 µm with the most significant field leakage. For the two M_1_ lasing modes with the highest Q-factors (*r_s_* = 8.5 µm and *r_s_* = 9 µm), we select *r_s_* = 9 µm as the fabricated shell domain airhole size. At this value, the Q-contrast is approximately 2.263, which is higher than the Q-contrast of about 2.013 observed at *r_s_* = 8.5 µm.

1. **Side Mode Suppression Ratio (SMSR) and power of Core-shell and Core-only BIC Lasers**

**
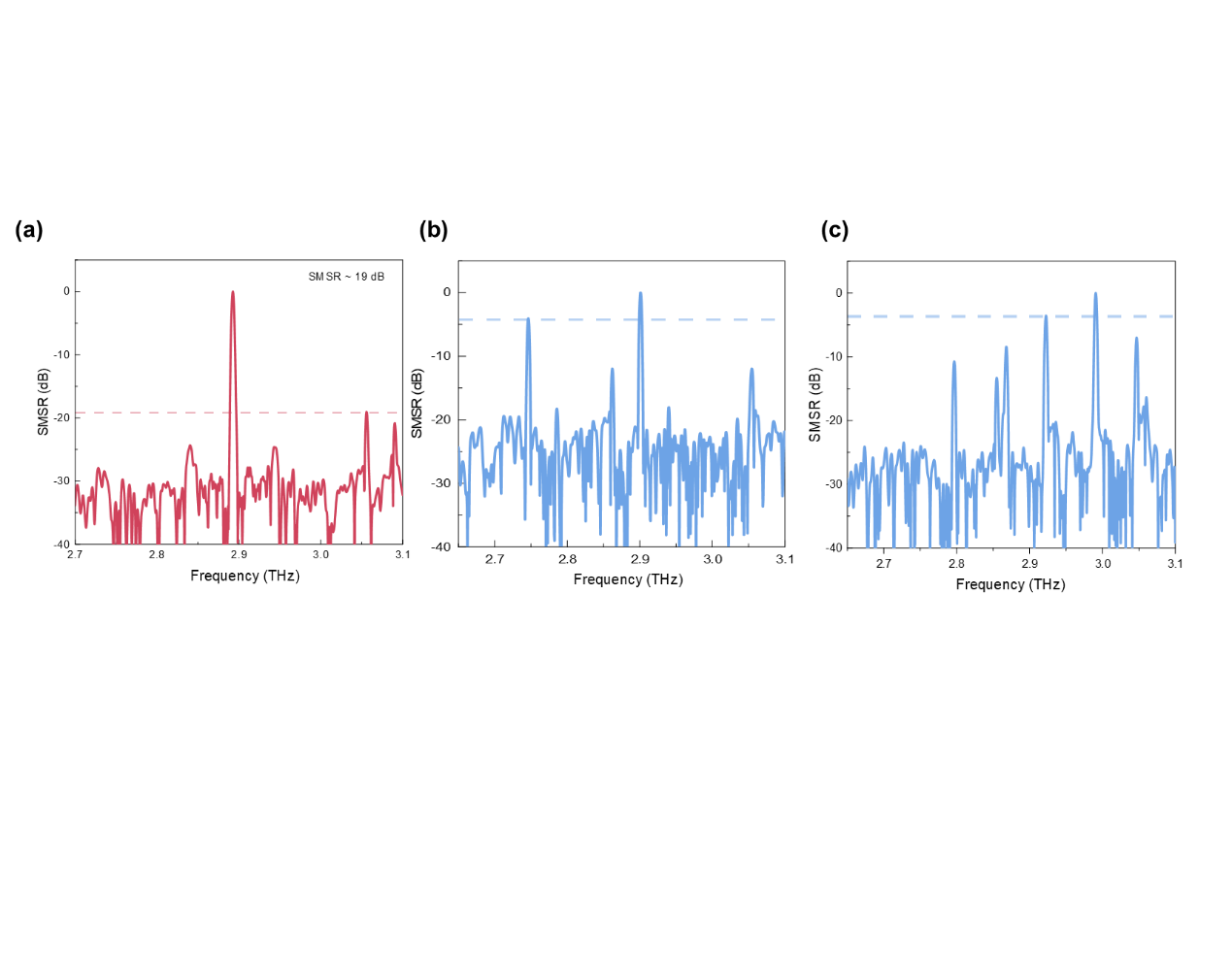
**

**Figure S8:** (a) SMSR for BIC laser of 9 by 9 periods of core domain with shell protection, whose spectrum and LIV plot is shown in Figure 4(b) and Figure 4(c). The strongest measured SMSR is around 19 dB at current density of 2.41 kA/cm^2^. (b) SMSR for BIC laser of 13 by 13 periods of core domain without shell protection, whose spectrum is shown in Figure 4(d). Highest SMSR ~ 4 dB happens at ~ 1.66 kA/cm^2^. (c) MSR for BIC laser of 15 by 15 periods of core domain without shell protection. Highest SMSR ~ 3dB happens at ~1.40 kA/cm^2^. Figure S6 shows that without shell domain protection, multiple lasing modes occur and SMSR is noticeably lower than SMSR for BIC laser with shell domain protection, even with larger core domain.

The power of the core-shell structure BIC laser is measured by a terahertz powermeter, Gentec-EO T-Rad with detector head of THZ9B-BL-DZ-D0 with a collection aperture of 0.9 mm in diameter. To enhance the collection efficiency, the laser emission was focused by one parabolic mirror, as shown in Figure S9(a). Sample with 21 by 21 periods of core domain and 5 periods of shell domain was measured. The pump pulse width was 1.00 µs and repetition rate were 10 kHz, which were also used for far-field measurement. The lock in frequency is 25 Hz. The device was measured in a dry nitrogen purging circumstance with an average power of 0.758 µW, resulting in a peak power of over 0.15 mW, as shown in Figure S9(b).


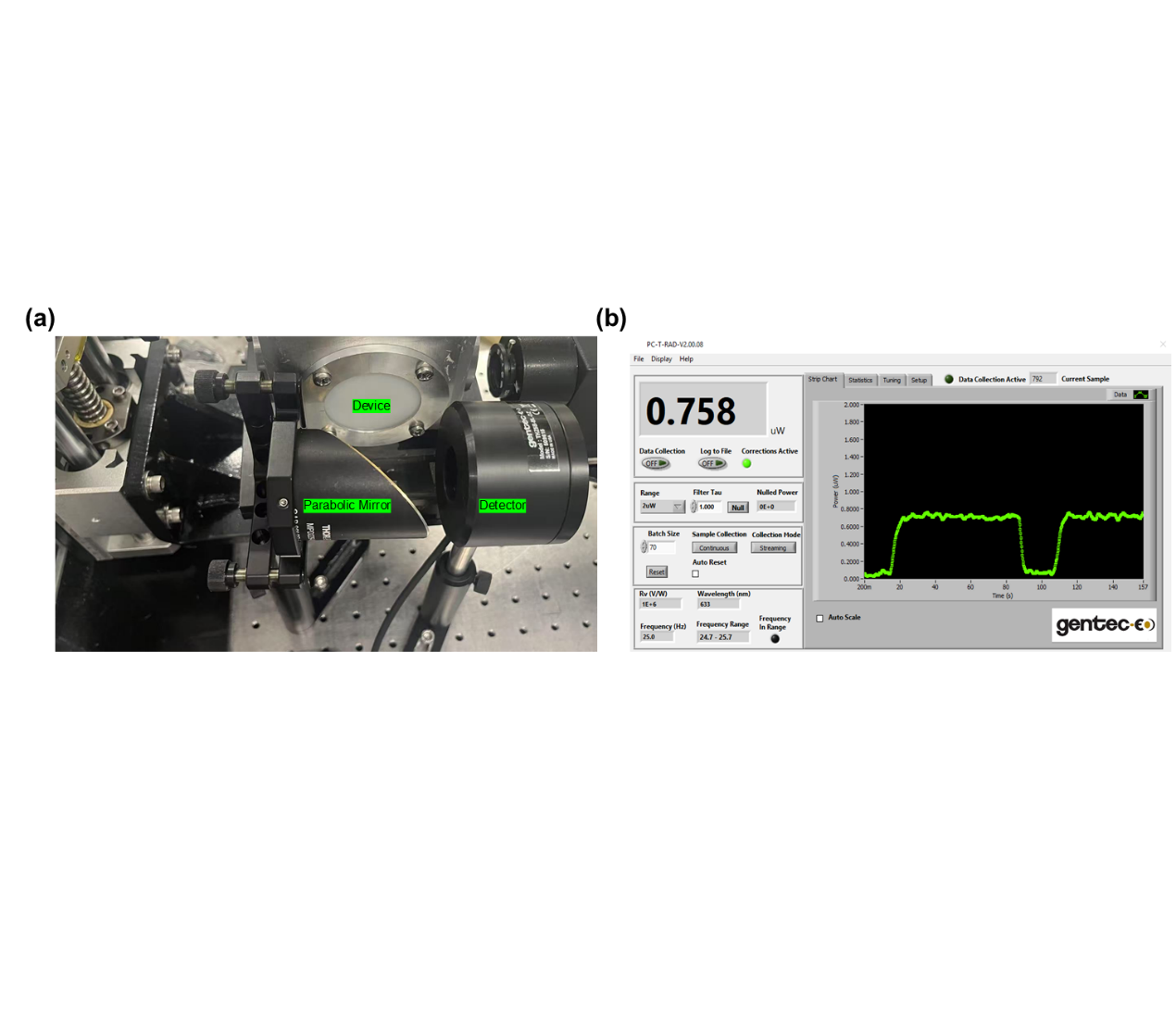


**Figure S9:** (a) Setup of power measurement. (b) The captured average power measurement software screenshot for the core-shell structure BIC laser device.


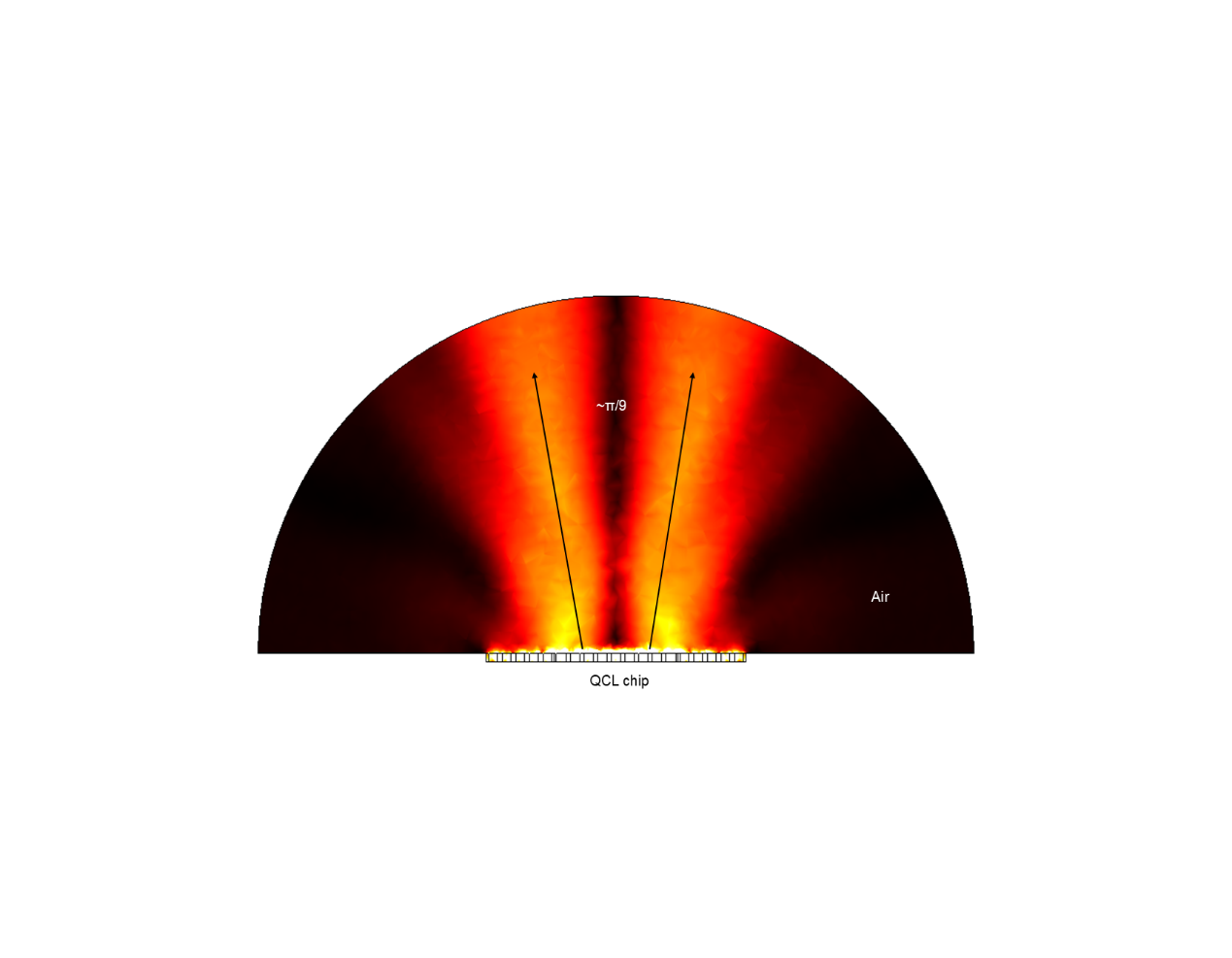


**Figure S10:** Simulated far-field pattern from the y = 0 plane, demonstrating a well-controlled divergence angle of approximately π/9.
